# Effect of glycerol plasticizer loading on the physical, mechanical, thermal, and barrier properties of arrowroot (*Maranta arundinacea*) starch biopolymers

**DOI:** 10.1038/s41598-021-93094-y

**Published:** 2021-07-06

**Authors:** J. Tarique, S. M. Sapuan, A. Khalina

**Affiliations:** 1 Advanced Engineering Materials and Composites Research Centre, Department of Mechanical and Manufacturing Engineering, Universiti Putra Malaysia (UPM), 43400 Serdang, Selangor Malaysia; 2 Laboratory of Biocomposite Technology, Institute of Tropical Forestry and Forest Products (INTROP), Universiti Putra Malaysia (UPM), 43400 Serdang, Selangor Malaysia; 3 Department of Biological and Agricultural Engineering, Universiti Putra Malaysia (UPM), 43400 Serdang, Selangor Malaysia

**Keywords:** Environmental sciences, Materials science

## Abstract

This research was set out to explore the development of arrowroot starch (AS) films using glycerol (G) as plasticizer at the ratio of 15, 30, and 45% (w/w, starch basis) using solution casting technique. The developed films were analyzed in terms of physical, structural, mechanical, thermal, environmental, and barrier properties. The incorporation of glycerol to AS film-making solution reduced the brittleness and fragility of films. An increment in glycerol concentration caused an increment in film thickness, moisture content, and solubility in water, whereas density and water absorption were reduced. The tensile strength and modulus of G-plasticized AS films were reduced significantly from 9.34 to 1.95 MPa and 620.79 to 36.08 MPa, respectively, while elongation at break was enhanced from 2.41 to 57.33%. FTIR analysis revealed that intermolecular hydrogen bonding occurred between glycerol and AS in plasticized films compared to control films. The G-plasticized films showed higher thermal stability than control films. The cross-sectional micrographs revealed that the films containing 45% glycerol concentration had higher homogeneity than 15% and 30%. Water vapour permeability of plasticized films increased by an increase in glycerol concentrations. The findings of this research provide insights into the development of bio-degradable food packaging.

## Introduction

The packaging plays a pivotal role in maintaining the food quality and regulating the interaction between the environment and food^1–3^. Due to magnificent versatility, mechanical and barrier properties, petroleum-based plastics mostly have wide applications in packaging industries^4–7^. Even though petroleum-based plastics have outstanding properties, they are also significant source of environmental issues due to their non-biodegradability. Hence, petroleum-based plastics are considered as the major factor, if not the only one, causing solid waste generation and build up in the environment. Petroleum-based plastics are non-biodegradable as well as originating from non-renewable sources^8^. These non-biodegradable petroleum-based polymers have caused growing concern about the wide usage of packaging due to the rapid depletion of petroleum reserves^9–15^. Owing to such concerns, the replacement of petroleum-based packaging with renewable and bio-degradable polymers materials have been stimulated since minimizing related environmental issues with natural polymers is vital^16–20^. In that regard, the agricultural resources have important biopolymers, such as polysaccharides and proteins to reduce the above-mentioned brief drawbacks of petroleum-based plastics^21–25^. Nowadays, natural compounds like proteins, lipids, and polysaccharides are utilized in making biodegradable and sustainable films^26–28^. According to Sartori et al.^29^, starch is one of the highest potentially polysaccharides owing to its potential to build a continuous matrix and low cost, plentiful, renewable, and available in various raw materials. Several researchers have vouched to the rising attention for developing bio-based packaging films by utilization of starch from many sources^30–38^. In this way, the arrowroot (*Maranta arundinacea*) rhizomes have emerged as powerful sources of starch and fiber.

Arrowroot (*Maranta arundinacea*) is mostly found in the tropical forest as a large perpetual herb and belongs to the *Marantaceae* family^39^. Arrowroot starch has excellent characteristics such as digestibility^40^, gelling ability^41,42^, as well as having the highest amylose content (40.86%)^43^, competing with corn starch (28–33%), wheat starch (30–32%), potato (18–20%), and cassava starch (16–19%), which are necessary for producing the films. Previous studies have documented that the film-forming properties of starch depend on the amylose content^44–47^, strong and stiff films are made by linking linear chains by hydrogen bonding. Hence, the high amylose content of arrowroot starch develops stronger films than other starch origins.

Nonetheless, Pelissari et al.^48^ reported that there are restrictions like brittleness and hydrophilic behaviour in starch-based films that directly impact the mechanical as well as barrier properties of the films, resulting in affecting the food packaging^49^. The restrictions, as mentioned above, can be overcome by adding the plasticizers, including glycerol, sorbitol, and polyethene glycol. Among the plasticizing agents, glycerol has been used as a plasticizer to produce starch-based films because of its compatibility with amylose^50^, which stimulates better mechanical properties through interfering with amylose packing by forces between molecules declines between the starch molecules. Plasticized-starch films showed more flexibility as well as feasibility than unplasticized films for different packaging applications^51^. Several studies demonstrated the efficacy of glycerol as a plasticizer with concentrations of 20–40% of the starch weight^52–55^. Mali et al.^52^, analyzed the impact of glycerol with the concentration of (0–40%) on tapioca, corn, and yam starches and showed that, regardless of starch types, the tensile strength decreased whereas the elongation at break improved with increasing the concentration of glycerol.

To now, even less attention is being paid to exploring the applications of arrowroot starch-based films as bio packaging. Therefore, in the current study, glycerol (0, 15, 30 and 45% w/w, starch basis) was incorporated in arrowroot starch using the solution casting technique. The impacts of glycerol plasticizer loading on physical, mechanical, thermal, environmental, structural, as well as barrier properties of arrowroot starch films were investigated.

## Materials and methods

### Materials

Native arrowroot starch was extracted from arrowroot tubers purchased from Norient Jaya Sdn Bhd Kuala Lumpur, Malaysia. The glycerol plasticizer (with 99.5% purity) was supplied by Evergreen Engineering & Resources Sdn. Bhd., Selangor, Malaysia. The solvent for preparing filmogenic solutions was used the distilled water.

### Film preparation and characterization

The arrowroot-based films were fabricated via the traditional solution casting technique in Biocomposite Lab (INTROP) University Putra Malaysia. Glycerol (G) was used as a plasticizer to investigate the effect of different glycerol concentration on AS films. The film making process mentioned below was referred to previous works of Sanyang et al.^56^ and Ilyas et al.^57^.

**Figure 1:**
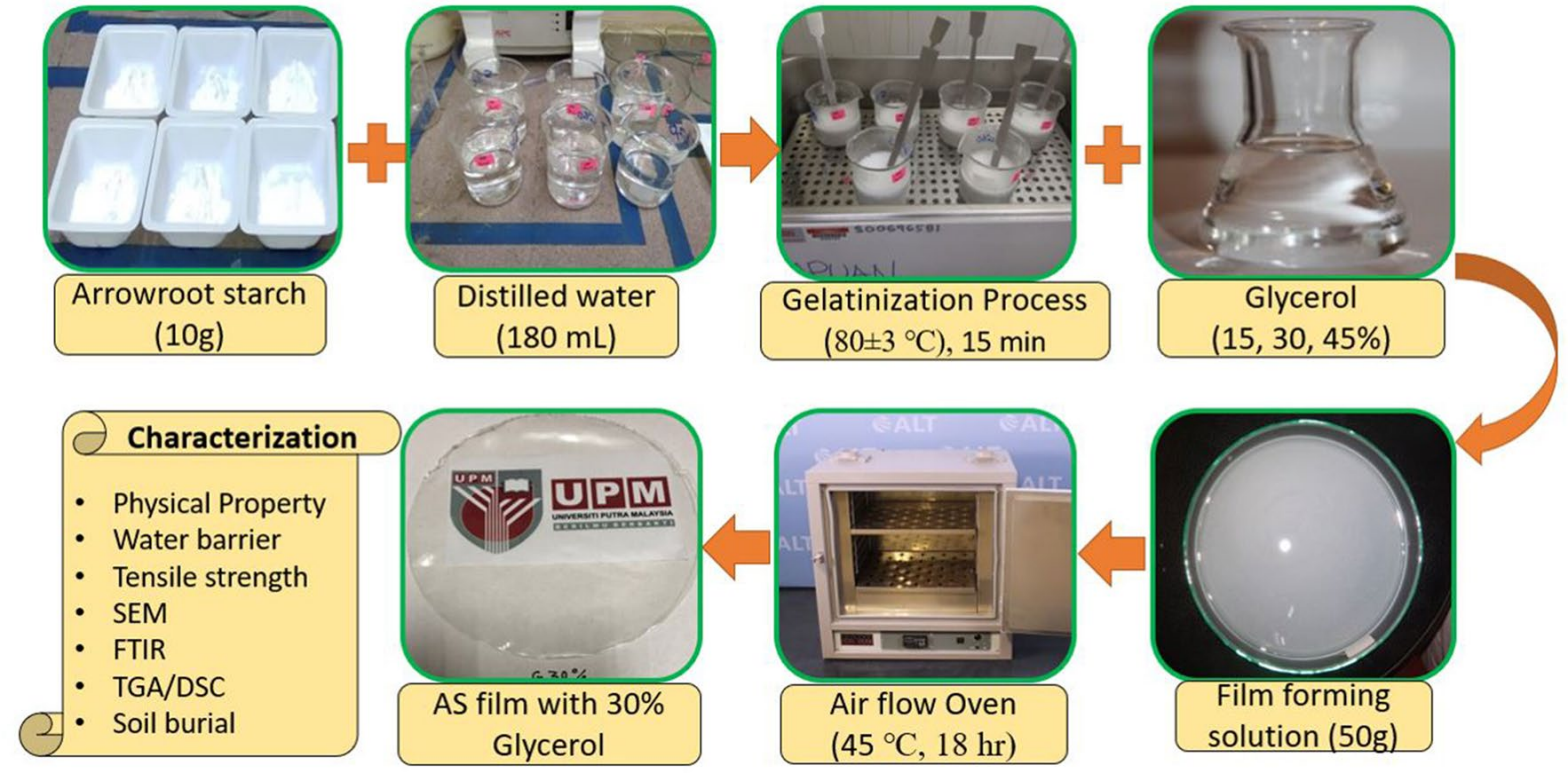
Flow diagram of film making process (Solution casting method).

At first, 10 g of arrowroot starches in gelatine form were made by applying heat at 80 ± 3 °C to the solution by slowing mixing in a thermostatic container. Intermolecular bonds of starch broke down during the gelatinization process in the presence of heat and water. Afterwards, the glycerol plasticizer (Evergreen Engineering & Resources Sdn. Bhd., Selangor, Malaysia) was incorporated with gelatinized solutions at 0, 15, 30, and 45% (w/w, starch basis), and it was maintained at the same temperature for an additional 5 min. After that, the film making solutions were let to cool and then cast in glass Petri dishes (diameter: 150 mm, 50 g) working as casting surface, which resulted in excellent appearance. This new film solution was placed inside the oven at (45 ℃) for 18 h to dry up properly. After drying, the Petri dishes were kept at room temperature for 24 h. The dry films were peeled out from the Petri dishes and stored at ambient conditions (25 ± 2 °C and 55 ± 1% RH) for a week before characterization. Figure 1 shows the flow process of film preparation.

### Physical characteristics

#### Thickness of film

An advanced remicrometre (Mitutoyo, Japan) was utilized to measure the film thickness with 0.001 mm sensitivity. In each film sample, five distinct locations were measured to determine the thickness of films and the mean value was estimated.

#### Film density

The density of film samples were determined utilizing Densimeter (Mettler-Toledo (M) Sdn. Bhd) and ASTM D792-00^58^. After that, the calculation of the preliminary dry matter of every sample was carried out. The film samples were weighed (m) before being immersed into the liquid of volume (*V*) solvent. The density (ρ) of the sample was determined by using Eq. (1). The test was performed in triplicate.

1 $${\uprho } = \frac{m}{V}.$$

#### Moisture content

A digital weighing scale was used to evaluate the moisture content of three replicates of each film sample. All the samples were weighed before (M_i,_ gram) andthen dried at 105 °C for 24 h and reweighed (M_f,_ gram). To calculate the moisture content of each film sample, Eq. (2) was used.

2 $${\text{Moisture}}\,{\text{content}} = \left[ {\frac{{W_{i} - W_{f} }}{{W_{i} }}} \right] \times 100.$$

#### FTIR analysis

FTIR spectrum and presence of functional groups of the samples were analyzed using a Thermo Fisher Scientific, model Nicolet 6700 (United States). A total of 16 scans at 4 cm^−1^ resolution varying from (4000 to 400 cm^−1^) consisted of the spectra samples.

### Water barrier properties

#### Water absorption

The water absorption analysis was conducted using ASTM D 570-98(1998)^59^. A temperature of 50 °C was used to dry the film samples for 24 h and the dried samples were placed in a desiccator for cooling ensuring consistent weight. After that, the films were weighed (*M*_*i*_, gram) and then submerged at room temperature in distilled water. A clean piece of cloth was used to wipe the immersed film samples and reweighed (*M*_*f*_, gram). The difference between initial and final recorded masses was calculated using Eq. (3).

3 $${\text{Water}}\,{\text{absorption}}\,\left( {{\% }} \right) = \left[ {\frac{{M_{f} - M_{i} }}{{M_{f} }}} \right] \times 100.$$

#### Film solubility in water

This test was conducted according to the method of Ilyas et al.^57^ with some modifications. A strip of (30 mm × 10 mm) dimension was cut out from each film sample in triplicate and dried in an oven at 105 °C for 24 h. To determine the initial dry weight (W_i_, gram) of each sample, the strips were weighted. Afterwards, each sample was immersed in a beaker containing 50 ml distilled water under constant magnetic stirring at 500 rpm and kept at room temperature (23 ± 2 ℃) for a period of 6 h. The film’s insoluble part was separated from the beaker and placed in an oven for 24 h at 105 ℃. To evaluate the weight of solubilized matter (W_f_, gram), the dried samples were weighed again. Equation (4) was used to calculate the WS of each sample.

4 $${\text{Solubility}}\,\left( {{\% }} \right) = \left[ {\frac{{W_{i} - W_{f} }}{{W_{i} }}} \right] \times 100.$$

#### Water vapour permeability (WVP)

The ASTM E96-95^60^ with some modifications was utilized to carry out the water vapour permeability. The test was performed in the appropriate conditions inside a desiccator with 53 ± 1% relative humidity at 23 ± 2 °C temperature. Firstly, 20 g of silica gel was filled into the cup (diameter: 30 mm). After that, the film samples were cut into a circular shape, stabilized at the opening of cylindrical cups, and left with a 3 mm vacuum to the uppermost part. Before leaving the cups in a relative humidity chamber (25 ℃, 75% RH), the test cups were weighed and recorded periodically until the equilibrium state was attained. Finally, test cups with the escalated weight were weighed and used in the calculation of WVP in the following Eq. (5).

5 $${\text{WVP}} = \frac{{\left( {{\Delta }m \times d} \right)}}{{\left( {A \times t \times P} \right)}}.$$

where Δm is the increased weight of the test cup (g), d is the film thickness (mm), A is the exposed area of the film (m^2^), t is the transmission time interval (s), and P is the partial pressure of water vapour on the film sample (Pa). The derived unit of the outcome is g s^−1^ m^−1^ Pa^1^.

### Thermal properties of biofilm

#### Differential scanning calorimeter (DSC)

The differential scanning calorimeter was used to investigate the DSC test by heating the sample in a temperature range of 35–200 °C at a 10 °C min^−1^ heating rate. The film sample was cut to 10 mm^2^ and conditioned at 25 °C and 60% relative humidity. Using these thermo-grams, the following factors were obtained: the onset temperature (T_0_) and the peak temperature (T_P_).

#### Thermogravimetric analysis (TGA)

To find the thermal stability of the film samples, the process of thermogravimetric analysis was done by using TA Instruments (Mettler-Toledo AG, Schwerzenbach, Switzerland). The test parameters were taken as the temperature was varied from 25 to 600 °C under a constant heating range of 10 ℃/min in a nitrogen gas medium. A 10 mg of the film sample was put and heated in the aluminium tray. The weight reduction versus temperature is illustrated in TGA analysis.

### Morphological properties of starch biopolymer

#### Scanning electron microscopy

The scanning electron microscope, SEM (Coxem-EM-30AX +) was employed to examine the morphology of the films at 5 kV of an acceleration voltage. The aluminium stubs were used to mount the film samples with double-sided adhesive tapes. Subsequently, samples were coated by a thin golden layer (0.01–0.1 µm) to prevent charging.

### Mechanical properties

**Figure 2:**
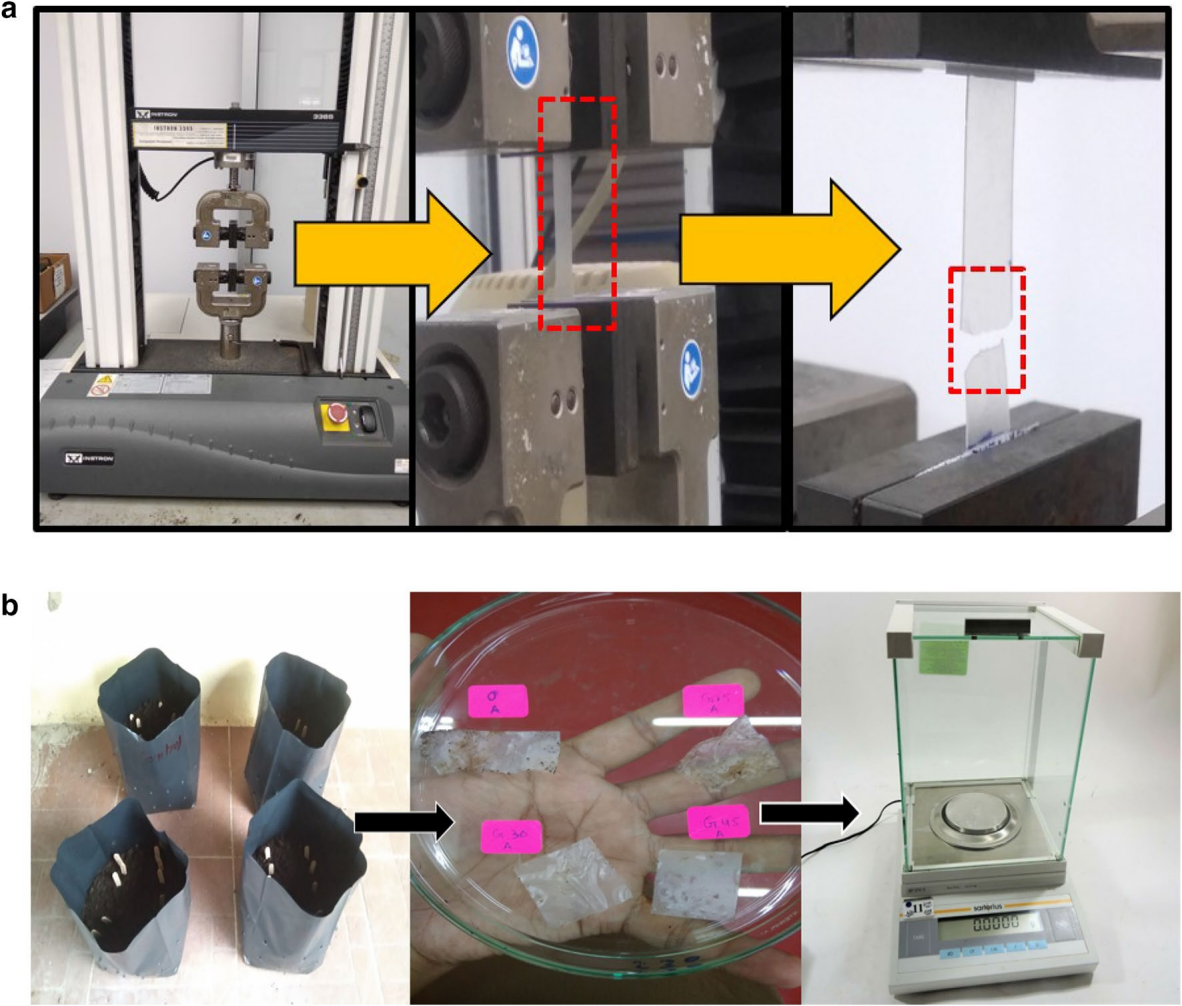
(**a**) Mechanical testing of arrowroot starch-based film samples. (**b**) Setup of the soil burial experiments.

The mechanical properties were evaluated utilizing the ASTM D882-02 (2002) standard^61^. The film samples were kept at the ambient conditions (23 ± 2 °C and 53 ± 1% RH) for 72 h. In the analysis of the mechanical properties of film samples, an Instron 3365 universal testing machine (High Wycombe, UK) with a loading cell of 30 kg was used as shown in Fig. 2a Initially, the film samples were cut into rectangular pieces with a scale of (70 × 10 mm) and the gauge length of samples were set at (30 mm). The specimens were held with 2 mm/min crosshead speed was applied to pull out the specimens. Deformation (mm) and force (N) of the specimen were then recorded. The evaluation of the mechanical properties was carried out using the average value of the measurements.

### Environmental analysis

#### Soil burial degradation

The biodegradability analysis of a film sample in soil was conducted by calculating the weight loss of control and G-plasticized arrowroot-based films buried in compost soil under restrained humidity conditions. The tests were performed in triplicate, where each film sample with 20 × 20 mm dimension was buried 100 mm underneath the surface of the soil. The characteristics of using soil were as displayed in Table 1. The samples were buried periodically at a time interval of 2, 3, 5, 7, 10, 12, 14, 16, 18, and 20 days and every sample were buried out from the compost simultaneously. After that, the sample was cleaned with water and dried in a vacuum oven at 65 °C to get a consistent weight^57^. Using Eq. (6), the weight loss of the sample was evaluated. The experiment setup showed in Fig. 2b

6 $${\text{Weight}}\,{\text{loss}}\,\left( {{\% }} \right) = \left[ {\frac{{W_{o} - W_{t} }}{{W_{o} }}} \right] \times 100$$

where (W_0_) prior burying weight and (W_t_) is post burying weight.

**Table 1:** Physicochemical properties of burial soil.

Moisture	45–55 (%)
pH	6.52
Carbon	30–40%
Nitrogen	1–1.5%
Phosphorus	1500–2000 ppm
Potassium	1500–2000 ppm
Magnesium	2000–3000 ppm

### Statistical analyses

The experimental data were statistically analyzed using Microsoft Excel 2016 and Origin 2019b software.

## Results and discussion

### The appearance of arrowroot starch biopolymers

**Figure 3:**
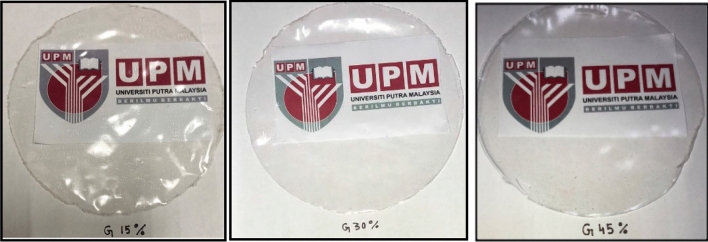
AS-based biopolymers developed utilizing distinct glycerol concentration.

Figure 3 presents the graphic images of the developed control and G-plasticized AS biopolymers, while Table 2 illustrates their visual presence. Without plasticizer, AS biopolymers were rigid, brittle, fragile, also wavy. They crash into bits, which made peeling and handling difficult. This finding might be due to the strong intramolecular hydrogen bonds, which gave less mobility to the macromolecular chains, which resulted in fragile and stiff surface broken films. This finding has also supported the observation of Sappakul et al.^62^ and Talja et al.^31^, who developed starch-based biopolymers with cassava and potato starch, respectively.

**Table 2:** The presence of control and G-plasticized AS biopolymers.

Sample	Glycerol (%)	Films appearance
AS	0	Translucent, wavy, brittle, rigid, and not peelable
G15	15	Translucent, no surface cracks, not brittle, not fragile, flexible, non-sticky, and easy to peel
G30	30	Translucent, more flexible compared to G15, slightly sticky
G45	45	Translucent, more flexible compared to G30, slightly elastic, sticky, and peelable

The incorporation of plasticizer to AS films made them flexible, homogeneous, and with even surface. It was observed that AS films with 45% glycerol was more flexible than 15% and 30% concentrations. Consequently, the flexibility of AS plasticized films was increased as increased the glycerol concentration. The increment in flexibility was due to plasticizer of the smaller molecular size, which allowed them to slide in the spaces between molecules of polymer chains, decreasing the strength of hydrogen bonds between molecules, therefore, boosting the movement of molecules. Consequently, increasing the concentration of plasticizer from 15 to 45% resulted in the weakened intermolecular hydrogen bond of AS films.

Owing to the smaller molecular weight of glycerol, it can be assumed that starch could interpose itself easily within the intra/intermolecular spaces. The plasticized AS biopolymers turn out to be peelable due to the strong bond between glycerol and AS.

### Film thickness

**Figure 4:**
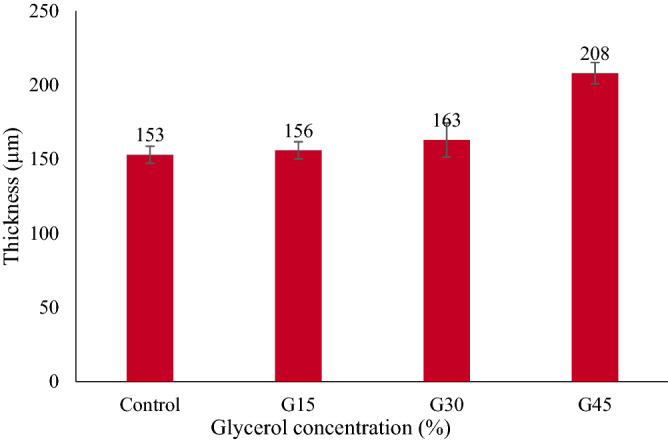
AS film thickness with different concentrations of glycerol.

There was a slight difference in the thickness of the control AS and G-plasticized films with 15 and 30% concentrations. Figure 4 shows the increase in film thickness from 156 to 163 µm in response to an increase in glycerol concentrations from 15 to 30%, while thickness was significantly increased from 163 to 233 µm with increasing glycerol concentration from 30 to 45%. This might be ascribed to plasticizers' role in upsetting and restructuring intermolecular polymer chain networks, converting all free volumes into the thicker film^63–65^.

### Water absorption

**Figure 5:**
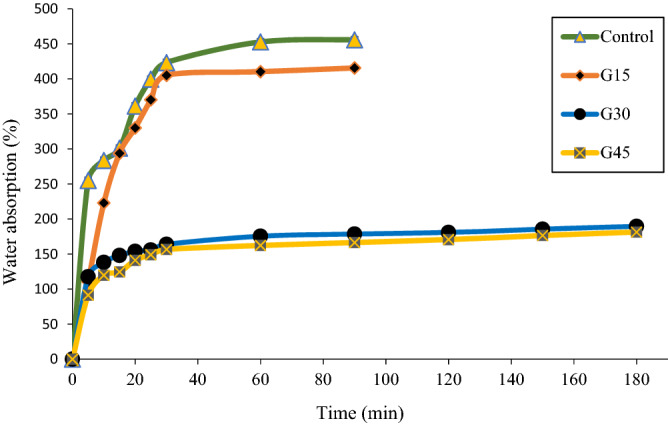
Water absorption of the control and G-plasticized AS films as a function of time.

The water absorption test result is presented in Fig. 5. This is a remarkable outcome that can be observed in Fig. 5, that after 30 min, films absorbed maximum water at room temperature. After 90 min, the control G15% films absorbed about 455% and 415%, respectively, while plasticized films with 30% and 45% absorbed about 178% and 166%, respectively. When immersion time reached 90 min, the control and all the films with different glycerol concentration started to dissolve in water except for the films plasticized with G30% and G45%. The films plasticized with glycerol 30% and 45% absorb less water compared to control and G15%. From the figure, it can observe that the water absorption of the plasticized AS decreased with the increase of glycerol concentration. This effect can be explained by the fact that glycerol formed a stronger hydrogen bond with arrowroot starch, preventing the water molecule from combining with the plasticizer or arrowroot starch. The findings also revealed that as the glycerol concentration increased, a stronger hydrogen bond between the plasticizer and arrowroot starch formed. Also, this might be summarized that the control AS film possessed lower water resistance compared to plasticized films due to their high hydrophilic nature. The water absorption tendency was attributed to the hydrogen bonds of starch formed in plasticizer molecules due to the presence of hydroxyl groups in plasticizer molecules as observed in FTIR structure analysis.

Other studies also reported that the water absorption of carbohydrate-based films decreased with increasing plasticizer concentration^66–68^. Sahari et al.^67^ reported that the rate of water absorption of plasticized sugar palm starch films linearly decreased (20.1, 17.1, 10.3 and 7.2%) with increased glycerol (15, 20, 30 and 40% w/w) respectively. As a plasticizer, such as glycerol, forms hydrogen bonds with starch, it destroys the current hydrogen bonds between hydroxyl groups in starch molecules. This is due to the forming of new hydrogen bonds between glycerol and hydroxyl groups. As a result, the plasticizing effect of the starch is enhanced, making it more difficult for water molecules to penetrate plasticized AS films.

### Film density

**Figure 6:**
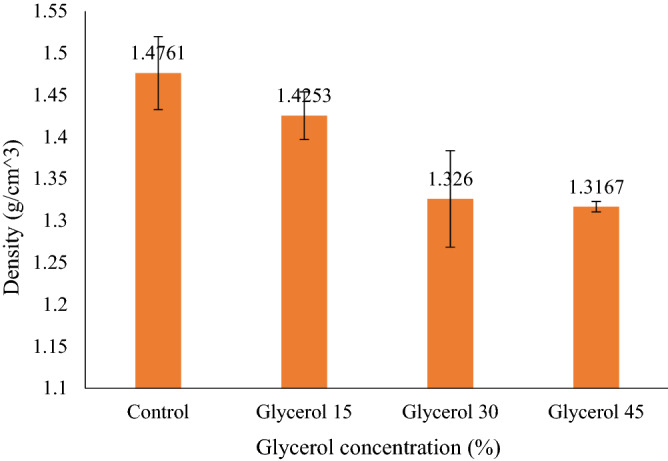
The density of AS films with different glycerol concentrations.

The incorporation of glycerol decreased the density of AS film from (1.476 g/cm^3^). Hence, all the G-plasticized films showed lower density relative to the control AS film. The impact of glycerol concentrations on the density of AS films are displayed in Fig. 6. Upon increasing the concentration of glycerol from 15 to 45%, there was a minor decrement in film density of AS/G- (1.425–1.316 g/cm^3^). By increasing the percentage of glycerol from 15 to 45%, the density of the films was slightly decreased. The decrease in density could be associated with the increased thickness (and volume) as a result of increased plasticizer content (refer to “Film thickness” section). This is in agreement with those reported by Razavi et al.^64^, Jouki et al.^65^, and Seyedi et al.^69^. The results of plasticized AS films with glycerol exhibited similar results as reported by Sanyang et al.^56^, who developed biopolymers using sugar palm starch and glycerol at the ratio of 15, 30, and 45% resulted in decrease the density from 1.493 to 1.407 g/cm^3^. Similarly, the findings of G-plasticized films are in agreement with those reported by Sahari et al.^67^ who used a dry processing technique (hot press) to plasticize sugar palm starch with glycerol (15, 20, 30, and 40%) resulted in decreased densities 1.46 g/cm^3^, 1.44 g/cm^3^, 1.40 g/cm^3^, and 1.32 g/cm^3^ respectively. Jouki et al.^65^ reported related effects on the density for the glycerol incorporation to the film of cress seed gum (CSG). Whereas, Nordin et al.^70^ reported that no significant difference was observed in density by adding glycerol in corn starch film. This can be ascribed to the change of film formulation that caused a simultaneous rise in the volume of the film, which increased the thickness of the film, hence, no significant difference in films’ density.

### Moisture content (MC)

**Figure 7:**
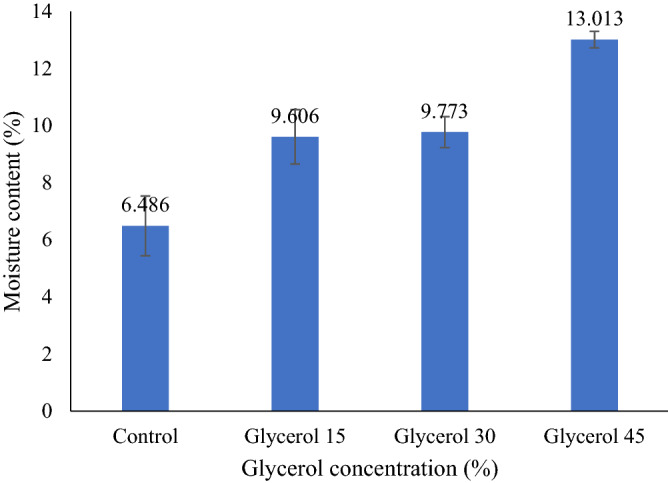
The moisture content of AS film with different glycerol concentrations.

MC of the control arrowroot starch film was observed to be lower than G-plasticized biopolymers. MC of all plasticized AS biopolymers enhanced significantly with enhancing plasticizer concentrations from 15 to 45%, as shown in Fig. 7. In general, the hydrophilicity of starch-based films was enhanced with an increase in the concentration of plasticizers. Consequently, several studies reported that the addition of plasticizers caused an increment in the moisture content of hydrocolloid films^56,71^. In this study, the moisture content of the G-plasticized films obtained were 9.84, 9.9, and 13.3% for the concentration of glycerol of 15, 30, and 45%, respectively, while Sahari et al.^67^ reported that the moisture content was reduced from 13.2 to 10.3% when increasing the glycerol concentrations from 15 to 40%. This phenomenon resembled the explanations of Cerqueira et al.^72^ that glycerol contained hydroxyl groups with a strong attraction with water molecules, which enabled the film to hold water and form hydrogen bonds within their structure.

### Film solubility

For selecting plastics for food packaging, film solubility in water is an essential criterion. Good water insolubility of films is vital for hydrophobic property to improving the shelf-life of food products^73^. Nevertheless, high water solubility can be essential for applying the edible coating to fresh and highly processed items.

**Figure 8:**
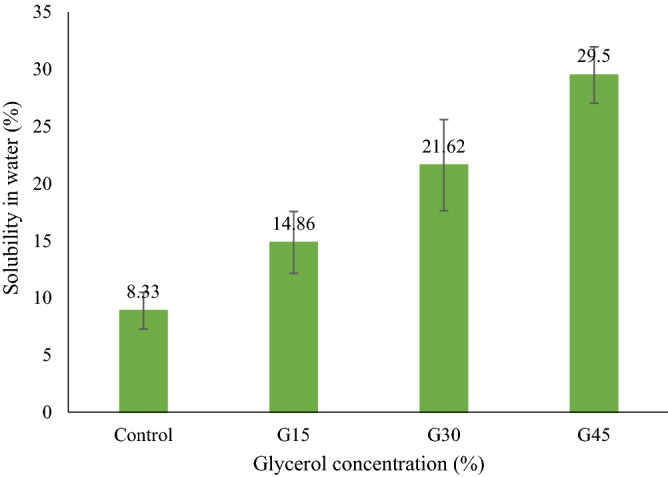
Films solubility in water.

Solubility in water test results for control and G-plasticized films are displayed in Fig. 8. The solubility of the control film was noticed to be lower than G-plasticized films. The solubility of plasticized AS films was significantly increased from 14.86 to 29.5%, with the introduction of the glycerol from 15 to 45%. These films showed less solubility compared to cassava starch as well as gelatin plasticized with glycerol, which showed solubility varying from 21.49 to 39.51%^74^. Basiak et al.^75^ also found that the solubility of films in water were about 14.52%, 30.16%, and 44.76% for potato, wheat, and corn starch, respectively. A higher solubility index for films might be due to higher amylose content. Sothornvit et al.^76^ described that the incorporation of plasticizers into biopolymers had modified the three-dimensional molecular organization of the polymer grid, reduced intermolecular attraction forces, and grew the free volume of the system. Besides, the grid of polymer became less dense, which enabled the water permeation in its structure and its solubilization.

Higher solubility of the film is a useful feature in case of ingesting packaging together with the food product, where this material is unsuitable for food packaging application, especially liquid food products.

### Water vapour permeability

WVP is used to measure the movement of water vapour via materials. In food packaging application, hydrophobicity is one of the most essential criteria for the selection of materials, because the performance of the product might be affected, either the product is water sensitive or not. Hence, in food packaging applications, the films need to have low WVP and decreased and prevented moisture transmission between the food and surrounding. Therefore, lowering the WVP of AS based films is crucial for their vital potential applications.

Effect of glycerol concentrations upon water vapour permeability of AS biopolymers can be seen in Table 3. The water vapour permeability of control AS films were not estimated because films were crushed due to the brittle nature of the materials.

**Table 3:** Effect of glycerol concentrations on thermal properties of control and G-plasticized AS films based on TGA, DTG and DSC characterizations and WVP of AS films blended with glycerol.

Sample	T_g_	Loss of moisture			First thermal decomposition			Mass residue at 600℃	WVP × 10^–9^ (g s^−1^ m^−1^ Pa^−1^)
	T_g_ (℃)	T_Onset_ (℃)	T_max_ (℃)	W_L_ (%)	T_Onset_ (℃)	T_max_ (℃)	W_L_ (%)	(%)	_
Control	117.38	86.78	100.04	12.92	294.55	314.03	75.09	12.18	–
G15	120.15	91.52	111.45	10.74	290.82	317.79	78.64	11.03	5.94 ± 0.4
G30	110.36	75.88	108.89	11.97	254.93	321.53	79.46	8.75	9.45 ± 0.1
G45	111.67	73.12	110.28	12.59	291.92	320.96	57.46	8.08	10.83 ± 0.1

As shown in Table 3, there is increase in water vapour permeability of AS starch films from 15 to 45 percent by weight. The obtained results showed that as the glycerol concentration was increased from 15 to 45%, WVP increment from 5.71 × 10^–9^ to 10.8 × 10^–9^ was observed. The plasticized AS films with 15% glycerol presented lower WVP compared to 30% and 45%. This might be attributed to excellent interaction between biopolymers leading to lower glycerol concentrations, causing a dense and high compact starch network and structure. As a result, the water vapour permeability values became lower. Adding more amount of glycerol upto 45% increase the mobility and flexibility of starch network chains as a result of structural change of the molecular interaction between starch and starch to a looser network. Therefore, the film matrices were less thick and the water vapour permeability values of films were finally improved.

Several studies reported the high values of WVP as starch-gelatine film^77^, oat starch film^78^, and potato starch films^31^. Usually, the hydrophilic part of the films allows the WVP. Hence, the WVP of films also depends on the hydrophilic/hydrophobic part of the film constituents.

### FTIR analysis

**Figure 9:**
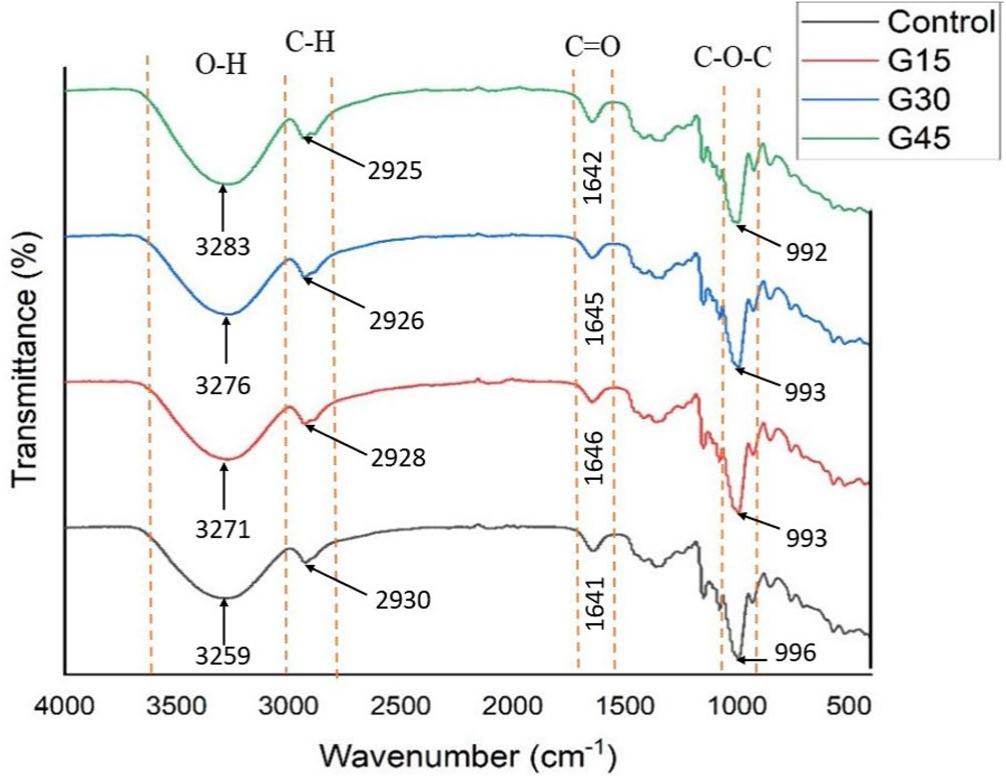
FTIR spectra of control and G-plasticized AS films.

Figure 9 presents the IR spectra of control and G-plasticized AS films. Based on Fig. 9, all the films showed similar spectra that can be attributed to the high content of starch on the film surface, which diminished the influence of glycerol addition. Wide bands in the range of 3000–3600 cm^−1^ were observed in all films, which correspond to the stretching vibration of O–H groups of starch and glycerol. Similar trends have also been identified^70,79–81^. The H-bonds identification between AS and glycerol occurred through the frequency shift of broad bands of O–H group in AS.

The peaks in the range of 2925–2930 cm^−1^ were ascribed to the (C–H) methyl group, as supported by the finding from Edhirej et al.^82^, Nazri et al.^83^ and Nordin et al.^70^. Meanwhile, a significant absorption peak in the range of 1642–1645 cm^−1^ was attributed to the hydroxyl group of absorbed water within starch films^80,84^. This peak might also be linked with O–H stretching vibration groups of glycerol as plasticizer. Different functional groups including C–O(H) and C–O–C also contributed to the absorption bands of 1105 cm^−1^ and 1150 cm^−1^, respectively. The band higher than 993 cm^−1^ was associated to C–O stretching vibrations of polysaccharide compound of starch and glycerol^85,86^. Similar peaks were noted on control as well as a plasticized film by Shirai et al.^87^, Sahari et al.^80^, Dai et al.^88^, Kurt et al.^79^, and Sanyang et al.^56^, in the ranges of 1020–995 cm^−1^, 1030–990 cm^−1^, 1082–1029 cm^−1^, 1150–1033 cm^−1^ and 1004 cm^−1^, respectively. Furthermore, the FTIR analysis revealed that adding plasticizer to AS-films ha dno significant effect on the chemical structure of the AS. This demonstrated that the molecular frames of the resulting AS-films were completely stable, with no significant chemical reactions occurring the plasticizer addition.

### Thermogravimetric analysis

**Figure 10:**
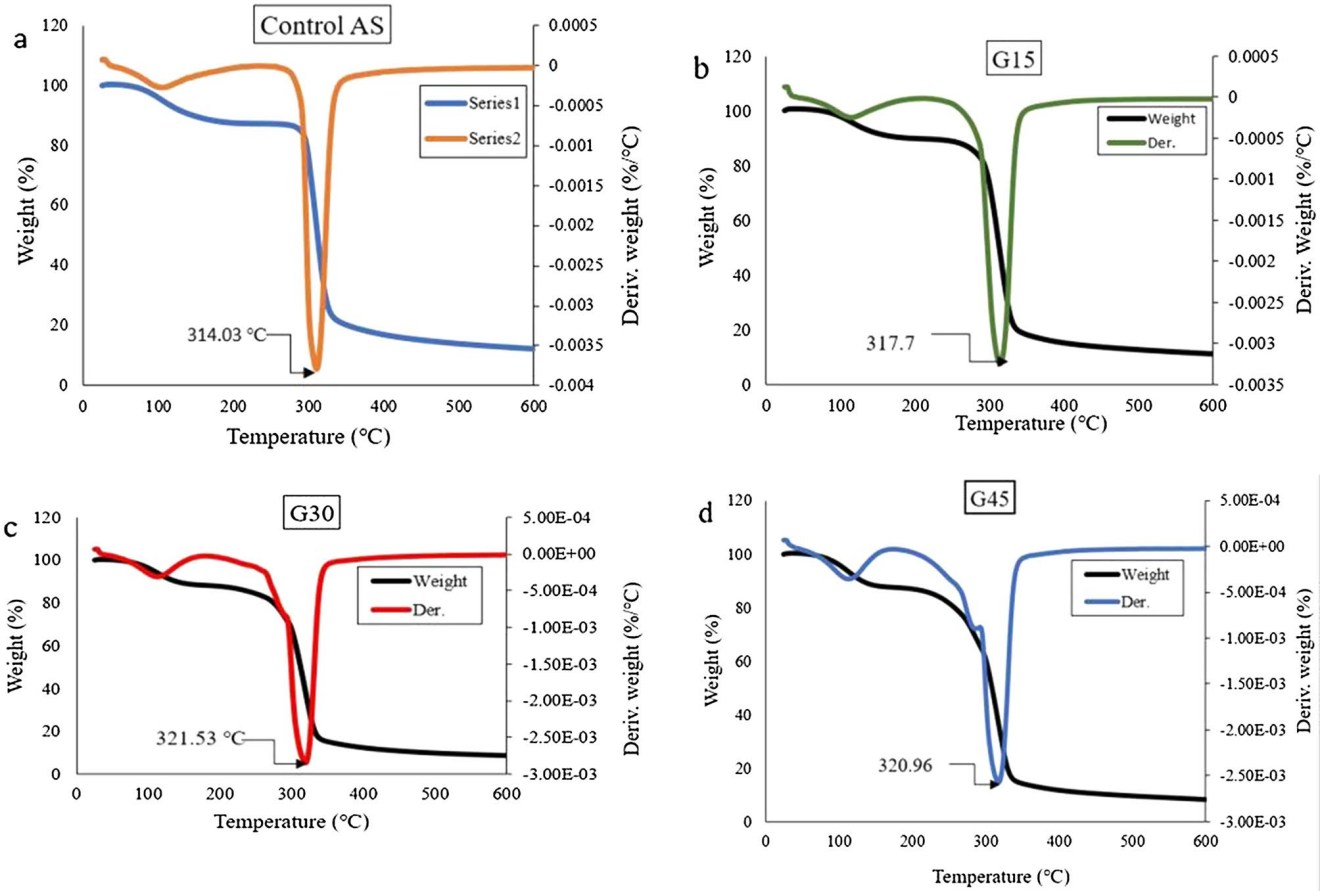
Thermo and derivative curves of control (**a**) AS, (**b**) G15, (**c**) G30, and (**d**) G45-plasticized films.

TGA has been employed to acquire the thermal degradation and stability of the control and plasticized AS films. The decomposition of films (mg) as a function of temperature (℃) is displayed in Fig. 10. There were three steps in the thermal degradation of the films, as shown in TGA and DTG curves. These three steps of thermal decomposition phenomena for most starch-based films were reported in previous studies^89–93^.

The first step of thermal decomposition of films happened below 100 ℃, which approximately 40–47 °C and was owing to loss of moisture content from the films. Simultaneously, this step was also related to loss of mass that can also be attributed to the loss of the weak bond of water molecules as well as low molecular weight compounds in films. All films, including control AS, experienced the same situation. By more increasing the temperature, the DTG of the control AS film displayed a sharp peak at 314.03 °C leading to a weight loss of approximately 75.15%, possibly due to the deterioration of the control AS saccharide rings^63^. At a temperature below 100 ℃, the control film displayed higher mass decomposition than plasticized biopolymers. This might be attributed to the fact that control AS film had higher water content compared to G-plasticized AS biopolymers.

The second step of thermal decomposition happened to vary of ~ 125–290 ℃, which related to the loss of glycerol compounds, including water molecules. These results of thermal decomposition were in good agreement with the results of other studies, Sanyang et al.^94^ and Ilyas et al.^57^, who studied on G-plasticized sugar palm starch. Likewise, Zhong et al.^95^ stated the decomposition temperature range of 150–280 °C for G-plasticized kudzu starch-based films. Further increment of temperature beyond 290 °C produced the highest rate of thermal decomposition, which was influenced by the radical weight loss of control film as well as G-plasticised films. From Fig. 10, the onset temperature of thermal decomposition of AS films occurred at approximately 300 ℃. This phenomenon might be ascribed to the removal of hydrogen functional groups, degradation, and depolymerization of the starch carbon chains polymer^96^.

It can be seen in Table 3 that the rise in the concentration of glycerol increases the rate of thermal deterioration of plasticized biopolymer relative to control film. For example, the percentage weight loss at 317.79 ℃, 321.53 ℃, and 320.96 °C was 78.64% for G15, 79.46% for G30 and 57.46% for G45 respectively, whereas 75.09% for control film at 314.03 ℃. In other words, increasing the glycerol concentration from 15 to 45% decreases the thermal resistance of AS films. This observation could be attributed to the glycerol–starch molecular interaction, which weakens the strong intermolecular bonds between starch molecules, lowering the thermal resistance of G-plasticized films^94^. Along with the TGA curve, the T_max_ of control and G-plasticized films were 314.03 °C and 321.53 ℃, respectively, indicating that the thermal stability of G-plasticized films was greater than that of thecontrol AS film. This can be attributed to the good interaction that occurs between the glycerol and the starch matrix, as evidenced by the FTIR spectra. This result was also supported by Nazri et al.^83^, Gutierrez et al.^97^, and Nordin et al.^70^, who found that the onset decomposition temperature of plasticized starch films was higher compared to that of control film due to strong contact between the plasticizer and the starch matrix. The final thermal occurrence at 600 shows the mass residue (%), with G45% having the lowest mass residue, followed by G15%, G30%, and control AS having the highest mass residue as shown in Table 3. This finding is most likely attributed to the volatilization of plasticizer from the films, which results in a lower mass residue of G-plasticized films than the control AS film.

### Differential scanning calorimeter (DSC)

In the DSC analysis of starch films, the transition glass temperature (T_g_) played an essential role in the thermophysical transition. According to Zhang et al.^98^, T_g_ is the temperature at which bounded amylose and amylopectin were loosened to lead a significant movement of the starch molecules.

Therefore, the glass transition temperature of control, as well as G-plasticized AS films, were obtained from DSC analysis are presented in Table 3. The results obtained from this study showed that the T_g_ of control AS films was 117.38 ℃. The incorporation of glycerol into control AS films caused to decrease in the T_g_. These results were in line with those of previous results that reported the reduction of T_g_ as the incorporation of plasticizers into starch-based biopolymers. Consequently, the movement of polymer chains increased as the polymer matrix became less compact^56,91^. While in the current analysis, the T_g_ values of AS films were significantly reduced as the percent of glycerol incorporated rose from 15 to 45%. In arrowroot starch films, the introduction of glycerol led to producing more hydroxyl groups as active sites, which might be conquered by water molecules. In the same context, Sanyang et al.^56^ reported that the starch-based films showed higher moisture content with higher glycerol content that led to lower glass transition temperatures (T_g_) values. Hence, this establishment between moisture content and T_g_ could be deemed a polymer inter-chain movement enhancer^52^. In their studies, these researchers also mentioned that the high content of glycerol allowed more interactions amongst glycerol-starch, glycerol-water, and glycerol-glycerol. Therefore, an increment in the free volume of films resulted in decreasing T_g_ values. Similarly, Chang et al.^99^ analyzed the impact of moisture and glycerol on the tapioca starch-based biopolymers and describing the findings that the high content of water, as well as plasticizer, caused lower T_g._

### Film morphology (SEM)

**Figure 11:**
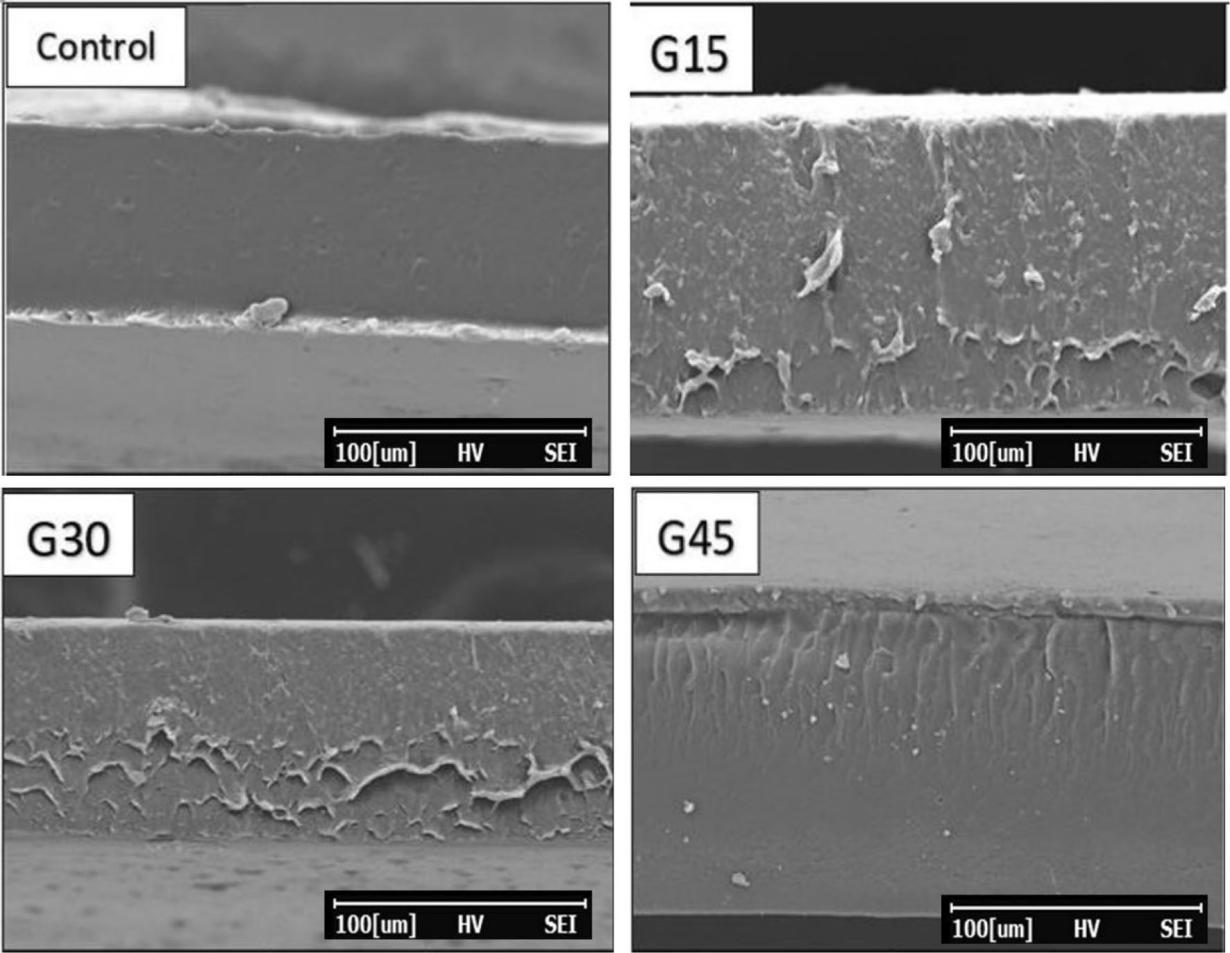
SEM images of the cross-section of control and G-plasticized AS films.

A scanning electron microscopy was utilized to analyse the cross-sectional of AS biopolymers. Figure 11 displays the Scanning electron microscopic images of the cross-section of films with 500 × magnification. In Fig. 11, G15, G30, and G45 films were cast from glycerol plasticizer with 15%, 30%, and 45% (w/w dry starch basis), respectively. AS films with 45% glycerol concentration presented higher homogeneity compared to 15% and 30% concentrations. The rough cross-section structures might be attributed to low interfacial adhesion between AS polymer and glycerol plasticizer, causing the weak forces in the tensile test, resulting in a decrement in tensile strength. A similar finding was observed by Martelli et al.^100^, who reported that a film developed from protein incorporated glycerol with different concentrations (1–9% w/w), in which the cross-section of the film with 9% showed more homogeneity. Likewise, Getnet et al.^101^ also describing the results that the biopolymers having 30% plasticizer were less homogeneous than 40% plasticized films. Cross-section micrographs of control and G-plasticized with 45% concentration exhibited more homogeneity. The homogeneity of these films attributed to their good structural integrity, and in addition, better mechanical properties might be expected^102^. These results demonstrated that films with high glycerol content were more homogenous compared to low glycerol concentrations.

### Tensile properties

**Figure 12:**
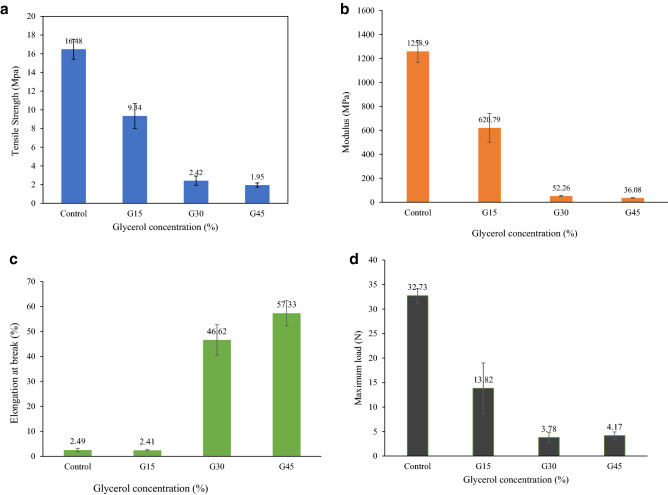
Effect of glycerol concentration on the (**A**) tensile strength, (**B**) tensile modulus, (**C**) elongation at break, and (**D**) maximum load of G-plasticized and control AS films.

Packaging material is required to sustain the integrity of the film to withstand extraneous forces. Mechanical properties such as tensile strength (TS) and elongation at break (EAB) of packaging material are important to avoid the stress that develops during storage, processing, and handling, whereas Young’s modulus (YM) of films indicates the stiffness of material^70^. Figure 12 shows the effects of increasing glycerol concentration on the TS, EAB, and YM of control AS biopolymers.

TS value of AS/G film decreased significantly from (16.15–9.34 MPa) when adding 15% glycerol into AS film, which was two times lower than control AS film. This happened due to the good compatibility of glycerol with starch, which permitted glycerol to interfere in between amylose packing within the starch matrix through H-bonding^50^. Consequently, the TS of the films was reduced significantly from 9.34 to 1.95 MPa as glycerol concentration increased from 15 to 45%. The phenomenon of high TS at low glycerol content might be that the dominance of strong intermolecular hydrogen bonds formed by starch–starch intermolecular interaction over starch-plasticizer attraction. Several studies have reported that TS of the starch-based biopolymers diminished when increasing plasticizer concentrations^32,65,71,103,104^.

Tang et al.^105^ also observed the reduction in TS by adding a plasticizer and reported a reduction in TS of corn starch/montmorillonite from 20 to 6 MPa as plasticized with 20% w/w glycerol. Similar findings were found by Rodriguez et al.^33^, where they observed a drop from 40 to 20 MPa in tensile strength of G-plasticized wheat starch biopolymer with 20% glycerol.

This trend might be described by the role of glycerol in reducing the strong intra-molecular attraction between the AS chains as well as supporting the formation of H-bonds between glycerol and AS molecules. Consequently, it diminished the TS of plasticized biopolymers by diminishing the hydrogen bonds between starch chains. Similar findings were observed by Razavi et al.^64^ and Muscat et al.^103^, who described that glycerol produces more significant TS reduction than other polyols. Hence, this trend might be attributed to the smaller molar mass of glycerol chains.

The G-plasticized AS films obtained higher TS values than the reported values by several studies, such as the film of corn starch–glycerol–stearic acid^106^, corn starch–glycerol–xylitol^107^, corn starch–glycerol^108^, corn starch–glycerol film^70^, cassava starch–glycerol film^109^, and cush–cush yam starch^97^.

### Elongation at break

EAB is the ability the extension of film length from the initial length to the breakpoint. In finding stretchability and flexibility, elongation (E%) plays a vital role. The required versatility of bio packaging films relies on their expected use and consequent transportation, handling, and storing of packaged foods and fruits.

Figure 12C clearly showed the effect of glycerol concentration (15–45%) on plasticized AS biopolymers' elongation had an inverse behaviour than their correspondent TS. As expected, the increase of glycerol concentration (15–30%) showed a significant enhancement in film elongation from 2.41 to 46.62%. Further glycerol increment from 30 to 45% also led to an increase in elongation compared to G30. On the other hand, the elongation of control and G15 films registered low values of 2.49% and 2.41%, respectively. Similar behaviour of film elongation was reported by several studies^31,52,62,79,103^. These results were observed owing to the decrease of the intermolecular bonds between amylose, amylopectin, and amylose-amylopectin of the starch matrix through the role of plasticizer, and hence, they were replaced by hydrogen bond which was present between starch molecules and plasticizer.

Such type of interference and re-enactment of molecular starch chains enabled to decrease the firmness and enhance the flexibility of films by enabling more chain mobility. It was reported that the mobility of molecular chains affected the elongation of polymeric^110^.

YM of films indicates the stiffness of the material, which opposes deformation against the applied force. The higher YM indicates a higher rigidity and stiffness of the film materials. As shown in Fig. 12B, YM for control AS film was the highest among the other films, which was 1258.9 MPa. The TS of films can also be related to their stiffness because the TS of the control AS films was highest and directly proportional to its YM. With glycerol incorporation, the films’ rigidity was significantly decreased to 620.79, 52.26, and 36.08 MPa, with the glycerol incorporation of 15, 30, and 45%, respectively.

### Soil burial degradation

**Figure 13:**
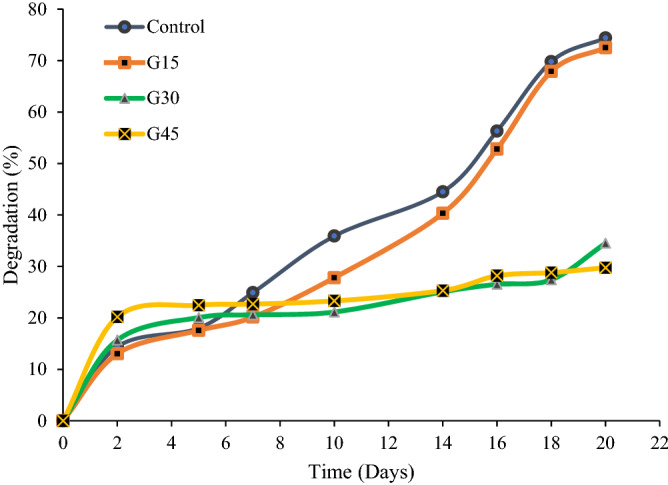
Degradation of control and G-plasticized AS films as a function of soil burial time.

Several factors such as fungi, bacteria, and microorganisms or other biological means play an essential character in the decomposition of materials. Initially, polymer starts to decompose as these microbial organisms interact with the biodegradable polymer^111^. The polymer transformed through the enzymatic or metabolic process by the action of these microbial organisms resulted in the polymers breaking down into smaller compounds, which has lower average molecular weight. The process of complete decomposition of material is known as mineralization^112^. In the present study, a soil burial degradation test was performed for the plasticized AS films with (0, 15, 30, and 45%) glycerol concentration and 20 days, as shown in Fig. 13.

As shown in Fig. 13, for the second day, the weight loss of film samples exhibited gradual increment and began to decelerate for consequent days with degradation time for both controls AS and G-plasticised film samples. At the end of 20 days, the weight loss of control AS and G15 were found at 74.4% and 77.5%, respectively while the G/AS film sample had lost 34.5% and 29.76% for G30 and G45, respectively. It was also calculated that the degradation rate of control and G15 films were 3.72 and 3.875%/day, while G30 and G45 degradation rates were 1.725 and 1.488%/day, respectively. It was also noticed that the decomposition of control AS film was higher than that of AS/G film at all given points of time. Gonzalez et al.^5^ ascribed this phenomenon to the close relationship between moisture content and microbial action of the soil. In other words, the rate of degradation increased as the water content increased in films. This finding was supported by the current study of water absorption results.

## Conclusions

In the current study, different concentrations of glycerol were used to investigate the effect on physical, structural, mechanical, thermal, environmental, as well as barrier properties of AS films. This study showed that the control AS films were brittle, fragile, and not peelable from the Petri dishes. Hence, the incorporation of glycerol as a plasticizer to AS film-forming solutions led to a decrease in the brittleness, fragility, and increase flexibility and peel ability of AS films. These results also demonstrated that the addition of glycerol to AS films resulted in the increment of the film thickness, moisture content, solubility in water, and WVP but, density and water absorption were reduced. The G-plasticized AS films demonstrated a significant decrement in TS and YM and increment in EAB values compared to control AS films. FTIR spectra analysis showed that intermolecular hydrogen bonding occurred between glycerol and AS compared to control films. The G-plasticized films showed the maximum decomposition temperature compared to the control AS film resulting in high thermal stability. Subsequently, G-plasticized films exhibited better barrier and environmental properties than other starch-based biofilms. Overall, the present study showed that different glycerol concentrations affected the physical, structural, mechanical, thermal, and barrier properties of G-plasticized films. In conclusion, the incorporation of glycerol led to improvements in the overall functioning of arrowroot starch films. In brief, the findings of this research provide insights into the development of biodegradable food packaging.

## Data Availability

The datasets generated during and/or analyzed during the current study are available from the corresponding author on reasonable request.
